# Preparative Separation of Sulfur-Containing Diketopiperazines from Marine Fungus *Cladosporium* sp. Using High-Speed Counter-Current Chromatography in Stepwise Elution Mode

**DOI:** 10.3390/md13010354

**Published:** 2015-01-09

**Authors:** Binbin Gu, Yanying Zhang, Lijian Ding, Shan He, Bin Wu, Junde Dong, Peng Zhu, Juanjuan Chen, Jinrong Zhang, Xiaojun Yan

**Affiliations:** 1 School of Marine Sciences, Laboratory of Marine Natural Products, Ningbo University, Ningbo 315211, China; E-Mails: gubin21314@gmail.com (B.G.); chenjuanjuan@nbu.edu.cn (J.C.); zhangjinrong@nbu.edu.cn (J.Z.); 2 CAS Key Laboratory of Tropical Marine Bio-resources and Ecology, South China Sea Institute of Oceanology, Chinese Academy of Sciences, Guangzhou 510301, China; E-Mails: zyy@scsio.ac.cn (Y.Z.); dongjunde@vip.163.com (J.D.); 3 College of Pharmacy, Jinan University, Guangzhou 510632, China; E-Mail: huahua20062008@126.com; 4 Ocean College, Zhejiang University, Hangzhou 310058, China; E-Mail: wubin@zju.edu.cn; 5 Key Laboratory of Applied Marine Biotechnology of Ministry of Education, Ningbo University, Ningbo 315211, China; E-Mail: zhupeng@nbu.edu.cn

**Keywords:** *Cladosporium*, high-speed counter-current chromatography, marine fungus, sulfur-containing diketopiperazines

## Abstract

High-speed counter-current chromatography (HSCCC) was successively applied to the separation of three sulfur-containing diketopiperazines (DKPs) (including two new compounds cladosporin A (**1**) and cladosporin B (**3**), and a known compound haematocin (**2**)) from a marine fungus *Cladosporium* sp. The two-phase solvent system composed of *n*-hexane-ethyl acetate-methanol-water at (1:1:1:1, v/v) and (2:1:2:1, v/v), in stepwise elution mode, was used for HSCCC. The preparative HSCCC separation was performed on 300 mg of crude sample yielding 26.7 mg of compound **3** at a purity of over 95%, 53.6 mg of a mixture of compounds **1** and **2**, which was further separated by preparative-HPLC yielding 14.3 mg of compound **1** and 25.4 mg of compound **2** each at a purity of over 95%. Their structures were established by spectroscopic methods. The sulfur-containing DKPs suppressed the proliferation of hepatocellular carcinoma cell line HepG2. The present work represents the first application of HSCCC in the efficient preparation of marine fungal natural products.

## 1. Introduction

The oceans are special environments with harsh conditions, such as high salinity, low-luminance, low temperature, high pressure, oligotrophic, *etc.* In order to adapt to the harsh environments, marine-derived fungi evolve unique metabolic and defense systems. Marine-derived fungi have been shown in recent years to produce a plethora of natural products with novel structures, which served as candidates for drug discovery [1,2,3].

Diketopiperazines (DKPs) constitute a large class of natural products that exhibit various biological properties. They were first discovered in 1880 and later studied by Emil Fischer [4]. Many of these compounds display diverse and noteworthy biological activities [5,6,7]. For example, Thaxtomin A acts as phytotoxin. Roquefortine C and acetylaszonalenin are mycotoxins. Albonoursin is antibacterial agents. Brevianamide S exhibits selective antibacterial activity against *Bacillus* Calmette–Guérin, suggestive of antitubercular potential. Ambewelamides A and B, phenylahistin, and verticillin A exhibit antitumor properties. Gliotoxin and sirodesmin PL have antibacterial, antiviral, and immunosuppressive properties. Haematocin is an antifungal DKP causing blight disease on ornamental plants, *Phalaenopsis* spp. and *Doritanopsis* spp. and first isolated from the culture broth of *Nectria haematococca* Berk. et Br. in 1999 by Suzuki and coworker [8]. Conventional isolation strategies for natural products involve multiple chromatographic steps, which were time consuming and resulted in substantial loss of samples due to irreversible absorption [8]. High-speed counter-current chromatography (HSCCC), being a support-free liquid-liquid partition method, eliminates irreversible adsorption of sample on to the solid support [9,10]. Because of these advantages, HSCCC has been widely used in the preparative separation of natural products [11]. In many cases, we can acquire compounds with high purity through one-step separation, while in other studies enrichment of sample were achieved. The potential of HSCCC in the efficient separation of marine natural products is being realized. In our recent reports, two macrolactin antibiotics and an antitumor ubiquinone were purified by HSCCC in one-step from crude extracts of two marine bacterium *Bacillus amyloliquefaciens* and *Pseudoalteromonas rubra*, respectively [12,13].

**Figure 1:**
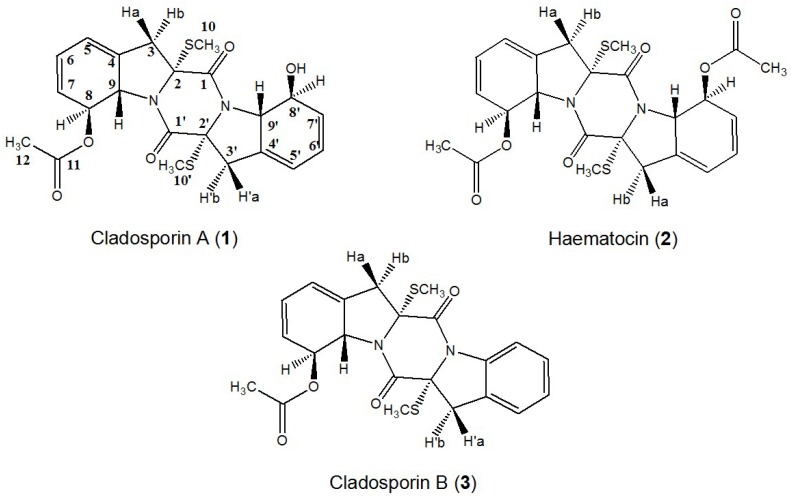
Chemical structures of cladosporin A (**1**), haematocin (**2**), and cladosporin B (**3**).

The aim of the present study was to develop a new and efficient method for the isolation and purification of two new sulfur-containing DKPs cladosporin A (1) and cladosporin B (3), together with haematocin (2) (Figure 1) from marine fungus *Cladosporium* sp. using HSCCC. 

## 2. Results and Discussion

### 2.1. HPLC Analysis of the Crude Sample

**Figure 2:**
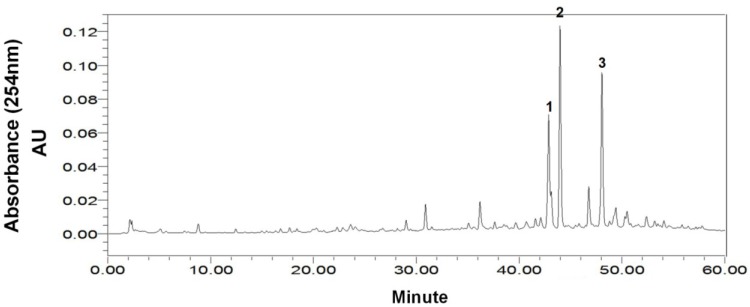
HPLC chromatogram of the crude extract from marine fungus *Cladosporium* sp. HPLC Conditions: column, Waters XBridge C18, 150 mm × 4.6 mm ID, 5 μm; column temperature, 25 °C; mobile phase, methanol and water at the gradient (methanol: 0–60 min, 10%–90%); flow rate, 0.8 mL/min; detection, 254 nm.

As shown in Figure 2, the HPLC chromatogram of the crude sample of *Cladosporium* sp. showed several compounds where the purities of target compounds **1**, **2**, and **3** were 12.6%, 20.6%, and 13.3%, respectively, based on HPLC peak area percentage.

### 2.2. Selection of the HSCCC Two-Phase Solvent System

The two-phase solvent system, *n*-hexane–ethyl acetate–methanol–water, has been widely applied in the separation of compounds with medium polarity by HSCCC [14]. The two-phase solvent system was selected according to the *K_D_* values of each target compound. In a previous review, Dr. Ito has demonstrated the golden rules in selecting optimum conditions for HSCCC. The two phase solvent system should satisfy the following requirements: (i) the suitable *K_D_* values for HSCCC are 0.5 ≤ *K* ≤ 2; (ii) to separate two compounds, the ratio of their *K_D_* values or the separation factor (α = *K_1_*/*K_2_*, where *K_1_* > *K_2_*) should be greater than 1.5; (iii) short settling time (<20 s) of two-phase solvent system is important for the retention of stationary phase; (iv) higher retention of the stationary phase provides better peak resolution [9]. 

Our preliminary studies were carried out to determine the *K_D_* values of the target compounds in the two-phase solvent system composed of *n*-hexane–ethyl acetate–methanol–water at various volume ratios (0.8:1:0.8:1, 1:1:1:1, 1.5:1:1.5:1, 2:1:2:1, 2.5:1:2.5:1, v/v/v/v) by HPLC and the results are summarized in Table 1. All the two-phase solvent systems had separation factors α > 3 for compounds **2** and **3** and α < 1.5 for compounds **1** and **2**, implicating that compound **3** could be well separated from compounds **1** and **2**, but compound **2** could not be separated from compound **1** in the five solvent systems tested. When the two-phase solvent system 1:1:1:1 was used, compounds **1** and **2** co-eluted as one peak and well separated from anterior peaks. Nevertheless, the two-phase solvent system at a volume ratio of 2:1:2:1 offered a suitable *K_D_* value (0.84) for compound **3**. Due to great difference between their solubility, the target compounds could not be separated using a single solvent system.

**Table 1:** The distribution constants (*K_D_*) of the target compounds at different ratio of volume in *n*-hexane–ethyl acetate–methanol–water solvent system (compound **1**, cladosporin A; compound **2**, haematocin; compound **3**, cladosporin B).

*n*-Hexane–ethyl Acetate–methanol–water	*K_D_*		
	Compound 1	Compound 2	Compound 3
0.8:1:0.8:1	1.80	2.01	6.37
1:1:1:1	1.37	1.57	5.22
1.5:1:1.5:1	0.67	0.76	2.39
2:1:2:1	0.22	0.24	0.84
2.5:1:2.5:1	0.11	0.13	0.52

To overcome this problem, stepwise HSCCC elution mode was developed to simultaneously separate compounds with large *K_D_* value difference [15]. This method has been successfully applied to the simultaneous preparative separation of three antioxidative resveratrol oligomers from a wild grape and two macrolactin antibiotics from a marine bacterium in our previous reports [12,16]. In present study, our strategy was to combine the selected solvent systems (1:1:1:1 and 2:1:2:1) successively in a single run, which involved two steps: the crude sample was first eluted with the solvent system at a volume ratio of 1:1:1:1 until compounds **1** and **2** were eluted out, which was then followed by the mobile phase of the second solvent system with a volume ratio of 2:1:2:1 until compound **3** was eluted.

### 2.3. Stepwise HSCCC Separation

**Figure 3:**
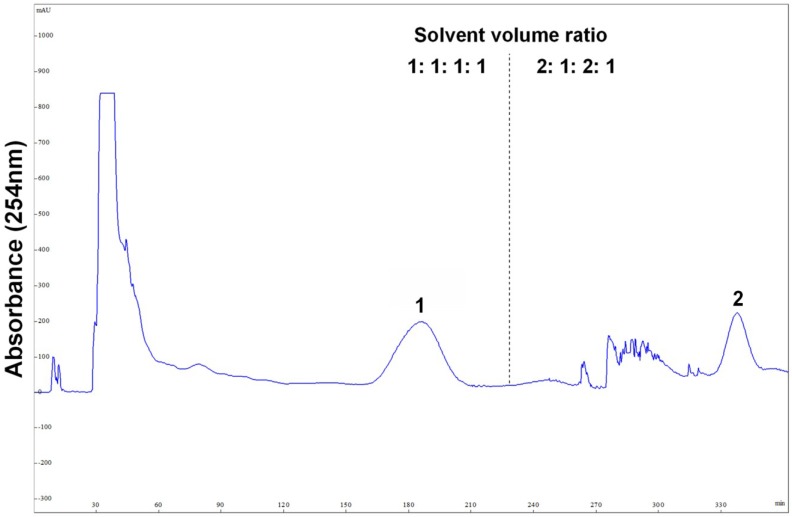
Preparative high-speed counter-current chromatography (HSCCC) separation of the crude sample from marine fungus *Cladosporium* sp. using stepwise elution with solvent systems A and B. Solvent system A: *n*-hexane–ethyl acetate–methanol–water (1:1:1:1, v/v), solvent system B: *n*-hexane–ethyl acetate–methanol–water (2:1:2:1, v/v); stationary phase: upper organic phase of solvent system A; mobile phase: 460 mL lower aqueous phase of solvent system A and 240 mL lower aqueous phase of solvent system B; column capacity, 300 mL; rotary speed, 900 rpm; column temperature, 25 °C; flow rate, 2.0 mL/min; detection, 254 nm; sample size, 300 mg of crude sample dissolved in 3 mL upper phase and 3 mL lower phase; retention of the stationary phase, 68%. Peaks: 1 = cladosporin A (**1**) + haematocin (**2**); 2 = cladosporin B (**3**).

The stepwise elution mode was applied for the preparative HSCCC separation of 300 mg of the crude sample with the following optimized condition: rotary speed, 900 rpm (recommended by the manufacturer); column temperature, 25 °C; flow rate, 2.0 mL/min; detection, 254 nm. As shown in Figure 3, the separation was started with the solvent system A (1:1:1:1), and after peak 1 was eluted (230 min at the dotted line in Figure 3), the mobile phase was switched to the lower phase of the solvent system B (2:1:2:1). Then peak 2 was well resolved and eluted in 350 min.

### 2.4. Preparative-HPLC Separation

**Figure 4:**
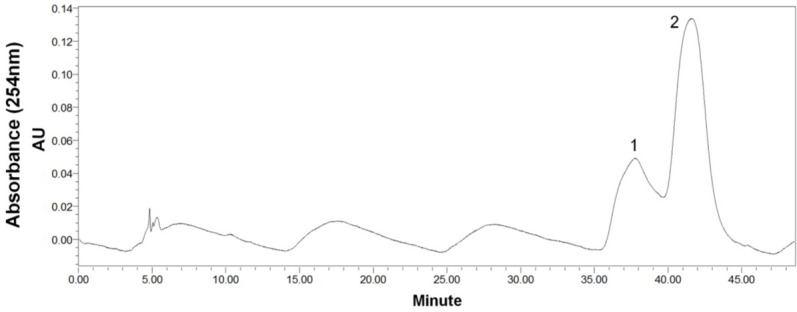
Preparative-HPLC chromatogram of peak 1 in HSCCC. Preparative-HPLC conditions: YMC-Pack ODS-A (Octadecyl-silica) column (250 mm × 10 mm ID, 5 μm, YMC, Japan); mobile phase: MeOH/H_2_O (55:45, v/v); flow rate: 3 mL/min. Peaks: 1 = cladosporin A (**1**); 2 = haematocin (**2**).

**Figure 5:**
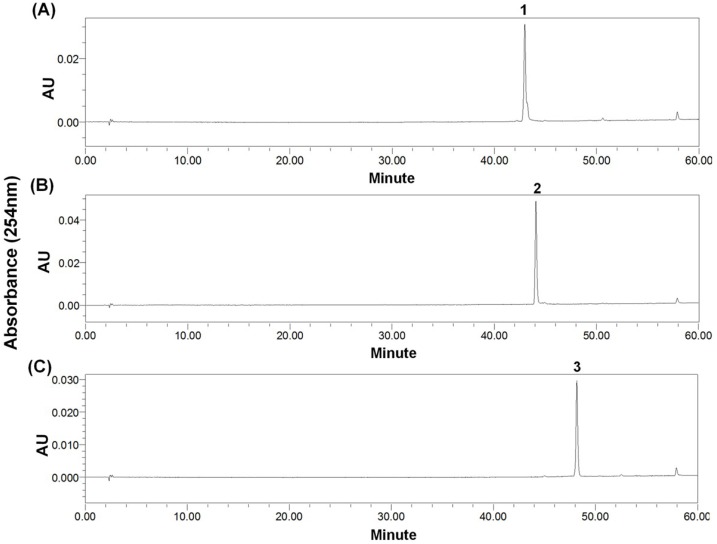
HPLC chromatograms. (**A**) Preparative-HPLC fraction of peak 1 (cladosporin A) in Figure 4; (**B**) Preparative-HPLC of peak 2 (haematocin) in Figure 4; (**C**) HSCCC fraction of peak 2 (cladosporin B) in Figure 3. HPLC conditions as shown in Figure 2. The wavy baseline was caused by an air-conditioner in the lab in summer, which periodically changed the temperature of the room.

After HSCCC separation, the peaks 1 and 2 were concentrated, yielding a mixture (53.6 mg) containing compounds **1** and **2**, and 26.7 mg of compound **3**, respectively. Then, the mixture (containing compounds **1** and **2**) was separated by preparative-HPLC (Figure 4), yielding 14.3 mg of compound **1** and 25.4 mg of compound **2**. Based on the HPLC analysis, the purities of peak 2 in HSCCC and peaks 1 and 2 in preparative-HPLC are all over 95% as shown in Figure 5.

### 2.5. Structural Identification and Cytotoxic Activity

**Figure 6:**
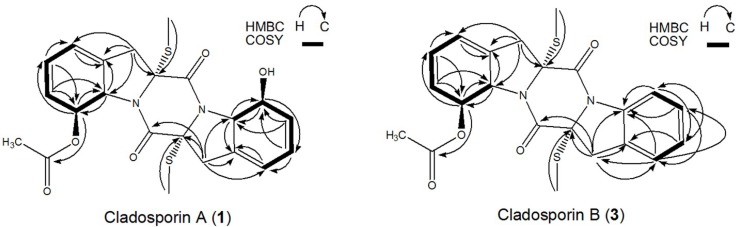
Key ^1^H-^1^H-COSY, HMBC correlations of compounds **1** and **3**.

Compound **2** was identified as haematocin (C_24_H_26_O_6_N_2_S_2_) by comparing with the NMR data given in reference [8]. The structure similarity of compounds **1** and **2** can be revealed from the UV spectrum, ^1^H NMR, ^13^C NMR, and related 2D NMR spectra. In addition, 42 ([-OCOCH_3_] − [-OH]) molecular weight difference (502 − 460 = 42) between compounds **1** and **2** suggests the existence of a hydroxyl group in compound **1** instead of a acetoxyl group in compound **2** (Figure 1), which can be further proved by ^1^H-^1^H COSY and HMBC experiments as shown in Figure 6. Therefore, the structure of compound **1** was identified as shown in Figure 1, and named as cladosporin A (**1**) (C_22_H_24_O_5_N_2_S_2_).

Compound **3** was determined to have a molecular formula of C_22_H_22_O_4_N_2_S_2_ from its HR-ESI-MS molecular formula. The similarities of molecular formula, UV spectrum, ^1^H NMR, ^13^C NMR, and related 2D NMR spectra of compounds **3** and **2** indicated that they have very similar structures. The ^1^H-^1^H COSY and HMBC experiments (Figure 6) revealed the existence of an intact benzene ring in compound **3** instead of a acetoxyl group appeared in compound **2**. Therefore, the structure of compound **3** was identified as shown in Figure 1, and named as cladosporin B (**3**).

Comparing the structure of these three compounds (Figure 1), they were from the same DKP biosynthetic pathway and derived from cyclic condensation of two l-Phe [5]. Furthermore, the relative stereochemistry of **1** and **3** was proposed as shown in Figure 1 according to the previously established relative stereochemistry of **2** [8]. 

Cytotoxic activity tests found that **1**–**3** exhibit moderate cytotoxic activities to HepG2 cell line, with IC_50_ at 21, 42, and 48 μg/mL respectively. Other biological activities including neuroprotective and antibacterial activities are under investigation. 

## 3. Experimental Section

### 3.1. Reagents and Materials

All organic solvents used for HSCCC were of analytical grade (Huadong Chemicals, Hangzhou, China). Reverse osmosis Milli-Q water (18 MΩ) (Millipore, Bedford, MA, USA) was used for all solutions and dilutions. Methanol used for HPLC analysis and preparative-HPLC was of chromatographic grade and purchased from Merck, Darmstadt, Germany. 

The marine fungus *Cladosporium* sp., based on 18S rDNA analysis, was isolated from a sediment sample collected from Yangshashan Bay, Ningbo, Zhejiang Province, China.

### 3.2. Apparatus

HSCCC was performed using a model TBE-300B high-speed counter-current chromatograph (Tauto Biotech Co. Ltd., Shanghai, China), which was equipped with three (polytetrafluoroethylene) multilayer preparative coils (ID 2.6 mm, total volume is 300 mL) and a 20 mL manual sample loop with the maximum rotational speed of 1000 rpm. The β values of the multilayer coil range from 0.5 at internal terminal to 0.8 at the external terminal. An ÄKTA prime system (GE, New Brunswick, NJ, USA) was integrated to pump the two-phase solvent system, collect the fractions, and measure the UV absorbance of the effluent at 254 nm. An HX 105 constant temperature regulator (Beijing Boyikang Lab Instrument, Beijing, China) was equipped to control the separation temperature. And a N2000 data analysis system (Institute of Automation Engineering, Zhejiang University, Hangzhou, China) was employed for data collection and analysis. 

The analytical HPLC and preparative-HPLC equipment was a Waters Alliance 2695 equipped with a Waters model 2996 diode array detector and Waters Empower System (Waters Co., Milford, CT, USA). ESI-MS analyses were performed using an electrospray ionization-quadrupole-time of flight mass spectrometry (ESI-Q-TOF MS, Waters, Milford, CT, USA). HR-ESI-MS data were acquired with a FT-ICR mass spectrometer (Bruker Daltonics, Fremont, CA, USA). The ionization conditions included a sheath gas pressure (N_2_) flow-rate, 25 L/min; auxiliary gas pressure (N_2_) flow-rate, 5 Abs; spray voltage, 2.5 KV; vaporizer temperature, 300 °C; and capillary temperature, 350 °C. NMR experiments were carried out using a Bruker AVANCE 500 MHz NMR spectrometer (Bruker, Fällanden, Switzerland) with inverse triple resonance probe at 25 °C.

### 3.3. Preparation of Crude Sample

The fungus *Cladosporium* sp. was cultivated in a seawater-based Zobell 2216E medium (Difco Laboratories, Detroit, MI, USA). A 45 L fermentation broth of the fungus cultivated at 150 rpm (inoculated into fermentation medium in a 1000 mL Erlenmerer flask) on a rotary shaker for 8 days, was centrifugated to remove microbial cell mass, which was then extracted with ethyl acetate. The extracts were combined and evaporated under reduced pressure at 40 °C yielding a crude sample (1.8 g). It was stored in a refrigerator (4 °C) for the subsequent HSCCC and preparative-HPLC separation.

### 3.4. Measurement of Partition Coefficients (K_D_) and Preparation of Two-Phase Solvent and Sample Solution

The distribution constants (*K_D_*) of each target compound in different two-phase solvent systems (*n*-hexane-ethyl acetate-methanol-water) were determined by HPLC as follows: a small amount (3 mg) of crude sample was added into a test tube containing 3 mL of pre-equilibrated two-phase solvent system. After shaking vigorously for 3 min, the mixture was separated by centrifugation at 3000 rpm for 3 min. Then, an aliquot of each phase (10 μL) was analyzed by HPLC. The *K_D_* value was defined as the ratio of the peak area of a given compound in the upper phase divided by that in the lower phase. 

Two-phase solvent system in the present study was prepared by mixing *n*-hexane-ethyl acetate-methanol-water (1:1:1:1, v/v) and (2:1:2:1, v/v). The solvent mixture was thoroughly equilibrated in a separation funnel at room temperature and the two phases were separated shortly before use. The sample solution was prepared by dissolving 300 mg of crude sample in a solvent mixture consisting of equal volumes of both upper and lower phases (3 mL for each phase).

### 3.5. Separation Procedure

HSCCC separation, the multilayer coiled column of TBE-300B (300 mL) was first entirely filled with the upper phase as stationary phase. The lower phase (mobile phase) was then pumped into the head end of the column at a flow rate of 2.0 mL/min, while the apparatus was run at a revolution speed of 900 rpm. After the mobile phase front was emerged and hydrodynamic equilibrium was established, the sample solution (6 mL) was introduced through the injection valve. Throughout the experiment, the separation temperature was controlled at 25 °C. The effluent from the tail end of the column was continuously monitored with a UV detector at 254 nm. The chromatogram was recorded immediately after the sample injection. Each peak fraction was collected according to the chromatogram. The retention of the stationary phase was computed from the volume of the stationary phase collected from the column after the separation was completed. 

The preparative-HPLC separation was performed as follows: YMC-Pack ODS-A column (250 mm × 10 mm ID, 5 μm, YMC Corporation, Kyoto, Japan); the solvent system consisted of isocratic 55% MeOH (aq.); the eluent was pumped at 3 mL/min for about 50 min (monitored at 254 nm) and 600 μL sample solution was injected through the sample injector. The peak fractions were collected according to the elution profile with compound **1** from 36 to 38 min, and compound **2** from 41 to 43 min.

### 3.6. HPLC Analysis and Identification of the Peaks

HPLC analyses of the crude extract, HSCCC peak fractions, and preparative-HPLC peak fractions were carried out on a Waters Alliance 2695 liquid chromatography system, equipped with a quaternary solvent deliver system, an autosampler, and a 2996 diode array detector (Waters, Milford, CT, USA). A reverse-phase Waters XBridge C18 column (150 mm × 4.6 mm ID, 5 μm, Waters, Milford, CT, USA) at 25 °C was applied for all analyses. Methanol–water was used as the mobile phase in gradient elution mode (methanol: 0–60 min, 10%–90%). The effluent was monitored at 254 nm and flow rate was 0.8 mL/min. The structural identification of each peak fractions was carried out by the analysis of MS, 1D and 2D NMR data.

Cladosporin A (**1**): white powder; [α]^25^_D_ −57.4 (*c* 0.3, MeOH); UV(MeOH) λ_max_ (log *ε*) 267.5 (3.18) nm; HR-ESI-MS (*m/z*): 483.1019 [M + Na]^+^ (calcd for C_22_H_24_O_5_N_2_S_2_Na, 483.1024); ^1^H NMR (CD_3_OD, 500 MHz): 6.11 (1H, br.d, *J* = 13.14 Hz, H-8), 6.02 (1H, m, H-7), 6.02 (1H, m, H-5), 5.94 (1H, m, H-5'), 5.91 (1H, m, H-7′), 5.66 (1H, m, H-6′), 5.58 (1H, m, H-6), 5.12 (1H, br.d, *J* = 13.14 Hz, H-9), 4.87 (1H, br.d, *J* = 13.44 Hz, H-8′), 4.80 (1H, br.d, *J* = 13.44 Hz, H-9′), 3.07 (1H, m, H-3b), 3.05 (1H, m, H-3a), 3.00 (1H, m, H-3′b), 2.98 (1H, m, H-3′a), 2.28 (3H, s, H-10), 2.15 (3H, s, H-10′), 2.07 (3H, s, H-12). ^13^C NMR (CD_3_OD, 125 MHz): 172.2s (C-11), 169.6s (C-1), 166.6s (C-1′), 135.8s (C-4), 134.6s (C-4′), 130.6d (C-6′), 128.5d (C-6), 126.5d (C-7), 124.7d (C-7′), 120.7d (C-5), 120.5d (C-5′), 76.8d (C-8), 75.9d (C-8′), 75.3s (C-2), 74.8s (C-2′), 69.6d (C-9′), 65.4d (C-9), 39.8t (C-3), 39.3t (C-3′), 21.4q (C-12), 14.7q (C-10), 14.4q (C-10′).

Haematocin (**2**): white powder; UV(MeOH) λ_max_ (log ε) 268.7 (3.24) nm; ESI-MS (*m/z*): 525.06 [M + Na]^+^; ^1^H NMR (CD_3_OD, 500 MHz): 6.11 (1H, br.d,* J* = 14.20 Hz, H-8), 6.01 (1H, m, H-6), 5.98 (1H, m, H-5), 5.57 (1H, br.d, *J* = 9.59 Hz, H-7), 5.12 (1H, br.d,* J* = 14.20 Hz, H-9), 3.01 (1H, br.d,* J* = 16.20 Hz, H-3b), 2.96 (1H, br.d, *J* = 16.20 Hz, H-3a), 2.25 (3H, s, H-10), 2.07 (3H, s, H-12). ^13^C NMR (CD_3_OD, 125 MHz): 172.3s (C-11), 167.1s (C-1), 136.3s (C-4), 128.4d (C-7), 126.7d (C-6), 120.5d (C-5), 76.9d (C-8), 75.8s (C-2), 65.8d (C-9), 40.6t (C-3), 21.4q (C-12), 14.7q (C-10).

Cladosporin B (**3**): white powder; [α]^25^_D_ −14.6 (*c* 0.4, MeOH); UV(MeOH) λ_max_ (log ε) 262.7 (2.96) nm; HR-ESI-MS (*m/z*): 465.0910 [M + Na]^+^ (calcd for C_22_H_22_O_4_N_2_S_2_Na, 465.0919), ^1^H NMR (CDCl_3_, 500 MHz): 8.02 (1H, br.d, *J* = 7.90 Hz, H-8′), 7.30 (1H, m, H-5′), 7.29 (1H, m, H-7′), 7.18 (1H, dd,* J* = 7.17, 7.15 Hz, H-6′), 6.21 (1H, br.d, *J* = 14.00 Hz, H-8), 6.00 (1H, m, H-5), 5.99 (1H, m, H-7), 5.62 (1H, m, H-6), 5.21 (1H, br.d, *J* = 14.00 Hz, H-9), 3.65 (1H, br.d, *J* = 16.90 Hz, H-3′b), 3.46 (1H, br.d, *J* = 16.90 Hz, H-3′a), 3.18 (1H, br.d, *J* = 16.20 Hz, H-3b), 2.94 (1H, br.d, *J* = 16.20 Hz, H-3a), 2.37 (3H, s, H-10), 2.21 (3H, s, H-10′), 2.14 (3H, s, H-12). ^13^C NMR (CDCl_3_, 125 MHz): 170.6s (C-11), 164.8s (C-1′), 162.9s (C-1), 140.2s (C-9′), 133.8s (C-4), 129.0s (C-4′), 128.3d (C-6), 127.9d (C-7′), 125.8d (C-6′), 125.2d (C-5′), 125.1d (C-7), 120.1d (C-5), 117.7d (C-8′), 75.4d (C-8), 74.1s (C-2), 72.4s (C-2′), 64.2d (C-9), 39.7t (C-3), 39.3t (C-3′), 21.4q (C-12), 14.8q (C-10), 13.4q (C-10′).

### 3.7. Cell Proliferation Assay

Human hepatocellular carcinoma cell line HepG2 was purchased from the Cell Bank of the Chinese Academy of Sciences (Shanghai, China), and cultured in DMEM with 10% fetal bovine serum at 37 °C in a humidified atmosphere containing 5% CO_2_. Cell viability was determined by cell counting kit-8 (Dojindo, Tokyo, Japan) assay as previously described [17].

## 4. Conclusions

Previously, we have shown that HSCCC is powerful technique in the separation of marine bacterial natural products [12,13]. In present work, an efficient separation of three sulfur-containing DKPs from marine fungus *Cladosporium* sp. was achieved by application of HSCCC for the first time. To the best of our knowledge, this was the first application of HSCCC in the separation and purification of marine fungal natural products. As “sample supply” has been one of the most challenging issues in marine drug discovery, development of efficient separation protocol for potential drug lead is essential. In this context, we proposed that HSCCC could be helpful in providing an alternative approach.
